# Formulation of *Gracilaria lemaneiformis* jelly using fuzzy mathematics evaluation^☆^

**DOI:** 10.1016/j.fochx.2025.102612

**Published:** 2025-06-03

**Authors:** Aoxue Hu, Shengjun Wu

**Affiliations:** a Jiangsu Key Laboratory of Marine Bioresources and Environment/Jiangsu Key Laboratory of Marine Biotechnology, Jiangsu Ocean University, Haizhou 222005, China; b Co-Innovation Center of Jiangsu Marine Bio-industry Technology, Haizhou 222005, China

**Keywords:** *Gracilaria lemaneiformis*, Jelly, Fuzzy mathematical model

## Abstract

As a new type of low-calorie and healthy dietary supplement, seaweed-based products are gaining popularity worldwide. In this study, *Gracilaria lemaneiformis* jelly was developed using *G. lemaneiformis* as the main ingredient. An orthogonal test combined with a fuzzy mathematical evaluation method was employed to optimize the formulation. The optimal parameters were determined as a material–liquid ratio of 6 %, vinegar addition of 0.2 mL, and heating for 10 min. The resultant jelly exhibited a smooth surface, compact structure, good transparency, fine texture, excellent elasticity, and a fresh seaweed flavor. This work provides a robust framework for optimizing gel-based foods using fuzzy mathematics, highlighting the potential of *G. lemaneiformis* in functional food applications.

## Highlights

• Fuzzy math optimizes 6 % ratio, 0.2 mL vinegar, 10 min heating.
• Smooth texture, elasticity, fresh asparagus flavor.
• Microbial safety meets China's GB 4789.2/3 standards.

## 1 Introduction

*Gracilaria lemaneiformis*, a red alga (Rhodophyta) belonging to the family Gracilariaceae, is a traditional medicinal and edible seaweed species native to temperate coastal regions. It thrives in clear waters at temperatures of 11–22 °C, exhibiting rapid growth in spring and autumn but susceptibility to erosion at extremes (> 25 °C or < 0 °C) (Cao et al., 2024). Recent studies have highlighted its rich composition of bioactive compounds, including sulfated polysaccharides, phycoerythrin, and dietary fiber, which contribute to its anti-inflammatory, antioxidant, and metabolic regulatory properties (Cai et al., 2024; Sen et al., 2023). These attributes position *G. lemaneiformis* as a promising candidate for functional food innovation, particularly in addressing modern health challenges such as obesity and cardiovascular diseases (Dang & Sun, 2023).

Gel-based foods, characterized by their elastic textures and water-retaining networks, have evolved significantly from traditional starch-based systems. Historically, starch retrogradation—a process where amylose and amylopectin reassociate into crystalline structures—has been central to forming gels like those derived from pea or sweet potato starch (Zhang et al., 2024). Such gels, while nutritionally versatile, often lack the bioactive profiles of marine-derived alternatives. Modern research has expanded this paradigm by incorporating novel ingredients, such as antioxidant-enriched konjac jelly (Jung & Joo, 2021) or seaweed-fortified gels (Kim, Kim, & Kim, 2019), demonstrating the potential of hybridizing traditional gel science with marine bioresources.

Despite these advancements, the application of *G. lemaneiformis* in gel-based foods remains underexplored. Existing studies on algal gels predominantly focus on conventional additives like carrageenan or agar (Yang, Li, Li, Guo, & Sun, 2021), overlooking the species-specific optimization of polysaccharide-rich seaweeds. This gap is critical, as *G. lemaneiformis* polysaccharides exhibit unique water-soluble and gel-forming properties that differ from commercial hydrocolloids (Guo, Yang, & Lin, 2009). To address this, our study integrates fuzzy mathematics—a computational method proven effective in sensory-driven food optimization (Xie, 2016; Zolfaghari, Mohebbi, & Najariyan, 2014)—with orthogonal experimental design. This dual approach enables systematic parameter screening (e.g., material–liquid ratio, heating time) while accounting for subjective sensory variables, such as texture and flavor.

By bridging traditional gel-forming principles (e.g., starch retrogradation mechanisms) with modern computational tools, this work aims to develop a novel *G. lemaneiformis* jelly that maximizes both sensory quality and functional benefits. The outcomes are expected to advance the utilization of understudied seaweeds in the food industry, offering a sustainable alternative to synthetic additives while aligning with consumer demand for natural, health-oriented products.

## 2 Materials and methods

### 2.1 Materials

The fresh *G. lemaneiformis* (taxonomically confirmed as *G. lemaneiformis* (Bory) *Greville*, *Rhodophyta*, *Gracilariaceae*) was harvested from the coastal waters of Lianyungang, Jiangsu Province, China, in October 2023. The seaweed was sun-dried to a moisture content of 12 ± 2 % (*w*/w) under controlled conditions (25–30 °C, 60 % relative humidity) and mechanically ground into uniform particles (0.5–1.0 mm) using a stainless steel grinder (Model DF-20, Dingli Machinery). The polysaccharide content of the dried *G. lemaneiformis* was determined via the phenol‑sulfuric acid method (Dubois, Gilles, Hamilton, Rebers, & Smith, 1956) and quantified as 45.2 ± 1.8 % (*w*/w) of the dry weight. All batches were stored in airtight containers at −20 °C to preserve biochemical stability.

### 2.2 Preparation

#### 2.2.1 Preparation process

The production of *G. lemaneiformis* jelly began with cleaning dry, mildew-free seaweed through triple rinsing in distilled water. The cleaned seaweed was mixed with water at varying material–liquid ratios (30:1000, 750, 600, 500, and 400 mL) and boiled at 100 °C for different durations (10, 15, 20, 25, or 30 min) under continuous mechanical stirring (200 rpm) to ensure uniform polysaccharide extraction. The boiled slurry was then filtered through a 100-mesh sieve to remove residual solids, after which specific volumes of vinegar (0.2, 0.4, 0.6, 0.8, or 1.0 mL) were added to adjust pH and stabilize the gel. The mixture was homogenized by agitation (150 rpm, 5 min), transferred into sterilized molds, and allowed to cool at room temperature (25 °C) for 2 h, followed by refrigeration at 4 °C for 12 h to complete gelation.

#### 2.2.2 Critical process parameters

The production of *G. lemaneiformis* jelly emphasized rigorous quality control through three critical stages. Dry, mildew-free *G. lemaneiformis* was selected as the raw material to ensure purity and safety. Impurities were subsequently removed via mechanical sieving and manual inspection, guaranteeing a clean substrate for processing. During boiling, the mixture was continuously stirred to prevent localized overheating and caramelization, thereby maintaining uniform heat distribution and preserving the structural integrity of the gel. These protocols ensured consistent product quality while minimizing microbial contamination risks.

#### 2.2.3 Sensory evaluation of jelly

The sensory quality of *G. lemaneiformis* jelly was evaluated by a trained panel (*n* = 10) under controlled conditions (22 °C, neutral lighting). Attributes—color (15 %), texture (25 %), aroma (25 %), and taste (35 %)—were scored using standardized criteria (Table 1), with triplicate sampling and palate cleansing between trials to ensure consistency. Instrumental analysis (colorimeter, texture analyzer) complemented sensory data for objective validation.

**Table 1:** Sensory evaluation criteria for *Gracilaria lemaneiformis* jelly.

Criteria	Excellent (15–20 points)	Good (8–14 points)	Fair (0–7 points)
Color	Yellow or light yellow, uniform, lustrous	Light yellow or colorless, no variegation	Dark yellow, dull, marked variegation
Taste & Texture	Elastic, non-sticky, moderate hardness	Moderate elasticity, slightly inferior taste	Weak elasticity, sticky, overly soft/hard
Structure	Smooth, compact, no bubbles/depression	Mostly smooth, minor bubbles/depression	Fragile, rough surface, semi-gel, severe depression
Flavor	Pronounced fresh seaweed aroma, umami-rich, no off-flavors	Mild seaweed aroma, slight vinegar notes	No seaweed aroma, sour or bitter aftertaste

### 2.3 Experimental design

#### 2.3.1 One-factor experimental design

The single-factor experiments were designed to isolate the effects of material–liquid ratio, heating time, and vinegar addition on the sensory quality of *G. lemaneiformis* jelly. For the material–liquid ratio test, dry seaweed (30, 60, 75, 100, or 150 g) was mixed with 1000 mL of water (corresponding to ratios of 3 %, 6 %, 7.5 %, 10 %, or 15 % *w*/*v*), boiled at a fixed temperature of 100 °C for 20 min under mechanical stirring (200 rpm), and filtered. For the heating time test, a fixed material–liquid ratio of 6 % (60 g/1000 mL) was boiled at 100 °C for varying durations (10, 15, 20, 25, or 30 min) with constant stirring. For the vinegar addition test, the 6 % mixture was boiled at 100 °C for 20 min, filtered, and then mixed with vinegar (0.2, 0.4, 0.6, 0.8, or 1.0 mL). All samples were cooled under standardized conditions (2 h at 25 °C, followed by 12 h at 4 °C) prior to sensory evaluation.

#### 2.3.2 Orthogonal experimental design

Through a single factor test, a better level was selected from the three factors, and the three-factor three-level orthogonal test was conducted. In addition, the final sensory score was determined on the basis of the fuzzy mathematical evaluation, and the optimal formula of *G. lemaneiformis* jelly was obtained. The factor levels are shown in Table 2.

**Table 2:** Orthogonal test factor levels of *Gracilaria Lemaneiformis* jelly.

Project	Assessment criteria	Points	Level
Color	Yellow or light yellow, uniform color, luster	15–20 8–14 0–7	excellent
	Light yellow or colorless, shiny, no variegated color		good
	Dark yellow, dull, dull, with marked variegated colors		secondary
taste	Elastic, non-stick teeth, soft and hard moderate	21–30 11–21 0–10	excellent
	General elasticity, non-stick teeth, taste slightly worse		good
	Weak elasticity, sticky teeth, taste too soft or too hard		secondary
texture flavor	Smooth texture, uniform and compact structure, block-shaped integrity, less bubbles, jelly-like, no depression Texture is more smooth, more intact block or slightly soft texture, a small number of bubbles, jelly-like, slightly depressed The structure is fragile, the surface is rough, the surface seeps water, the semi-gel shape, the serious depression	21–30 11–20 0–10 15–20 10–16 0–9	excellent good secondary
	Fresh, fresh, *Gracilaria Lemaneiformis* umami, no vinegar flavor *Gracilaria Lemaneiformis* has a light flavor and no odor No *Gracilaria Lemaneiformis* flavor, a strange smell		excellent good secondary

### 2.4 Establishment of a fuzzy mathematical model of the sensory score of *G. lemaneiformis*

#### 2.4.1 Evaluation object

The set of evaluation objects referred to the set of samples for the orthogonal test of the *G. lemaneiformis* jelly, X = {X_1_, X_2_, X_3_, …, X_9_}.

#### 2.4.2 Evaluation factors

Four items were taken as factors influencing the sensory evaluation of *G. lemaneiformis* jelly, which were T = {T1, T2, T3, T4}, where T1 represents the appearance and color of *G. lemaneiformis* jelly; T2 represents the taste and texture of *G. lemaneiformis*; T3 represents the tissue status of *G. lemaneiformis*; T4 represents the gelatinous effect of *G. lemaneiformis* jelly.

#### 2.4.3 Evaluation rating set

*G. lemaneiformis* jelly was divided into three grades, A = {A1, A2, A3}. A1, A2 and A3 indicate excellent, good and secondary, respectively.

#### 2.4.4 Hierarchical weighting analysis

The weighting coefficients for sensory attributes of *G. lemaneiformis* jelly were determined through a structured 0–4 scoring system, implemented by a trained panel (*n* = 10) with expertise in food texture evaluation. Attributes—appearance (15 %), color (15 %), taste (35 %), texture (25 %), and gel integrity (10 %)—were systematically scored under standardized conditions. Weight coefficient was calculated as W = {W1, W2, W3, W4}, and ∑Wi = 1 (m = 4). Furthermore, the result was compared with the total score.

#### 2.4.5 Matrix analysis of sensory scores

The sensory evaluators were scored and evaluated in accordance with the sensory evaluation table. After obtaining the results, the number of times was divided by the number of evaluators, that is, the fuzzy matrix R was obtained, and the sensory quality comprehensive evaluation table of the sample was obtained as *X* = *W* × *R*. In determining the advantages and disadvantages of the sample, three grades of 95, 80 and 65 were assigned, and then the comprehensive evaluation results of sensory quality were multiplied by the corresponding grade scores to obtain the specific sensory score of the sample (Xie, 2016).

### 2.5 Quality evaluation of *G. lemaneiformis* jelly

The total number of colonies of the product was tested in accordance with GB 4789.2–2016 ‘Food Safety National Standard Determination of the Total Number of Colonies in Food Microbiology Inspection’ (National Health Commission of the People's Republic of China, and State Administration for Market Regulation, 2022). Coliform bacteria were detected in accordance with the most probable number counting method in GB 4789.3–2016 ‘Food Safety National Standard Food Microbiology Test Coliform Count’ (National Health Commission of the People's Republic of China, 2016).

## 3 Results and analysis

### 3.1 Analysis of one-factor test results

#### 3.1.1 Effects of solid–liquid ratio on *G. lemaneiformis* jelly sensory quality

**Fig. 1:**
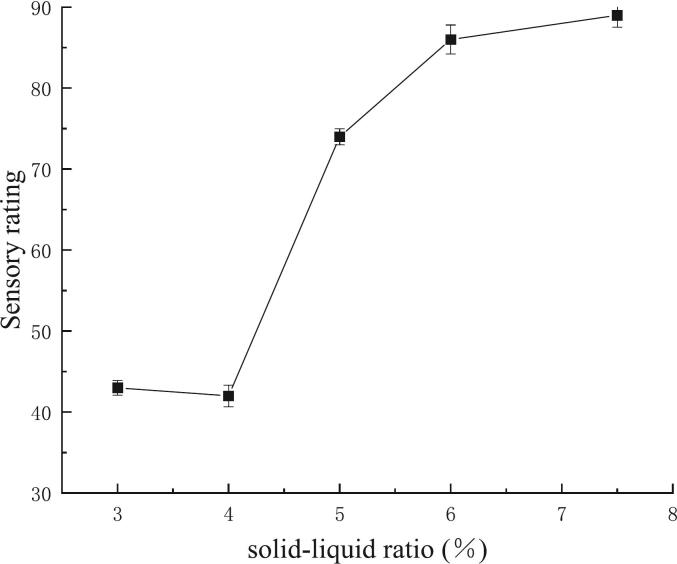
Effects of material–liquid ratio on *Gracilaria lemaneiformis* jelly sensory quality.

The unshaped and water-oozing samples observed at low material–liquid ratios (3 % and 4 %) (Fig. 1) can be attributed to insufficient polysaccharide concentration to form a stable gel network. *G. lemaneiformis* polysaccharides (GLPs) require a critical concentration to initiate intermolecular hydrogen bonding and hydrophobic interactions, which stabilize the three-dimensional gel matrix (Guo et al., 2009). At ratios below 5 %, GLPs are too dispersed to form continuous junction zones, resulting in weak gel strength and syneresis (water expulsion). This aligns with studies showing that agarose-based gels similarly require minimal polysaccharide concentrations (≥5 % *w*/*v*) to establish cohesive networks (Zhang et al., 2024).

#### 3.1.2 Effects of heating time on *G. lemaneiformis* jelly sensory quality

**Fig. 2:**
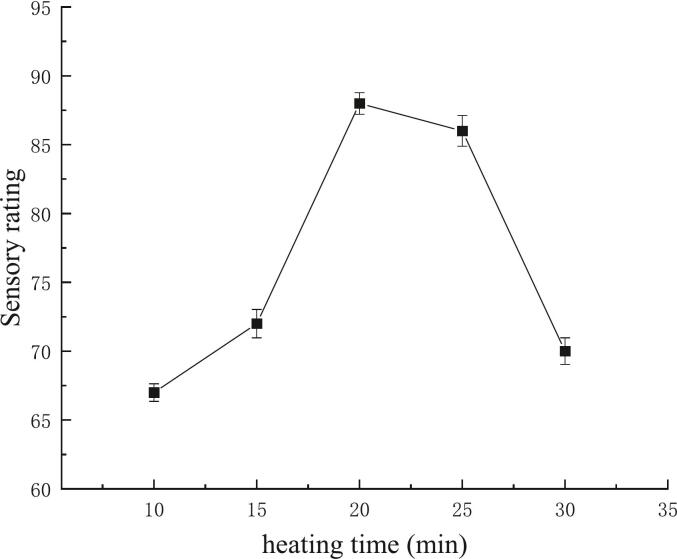
Effects of heating time on *Gracilaria lemaneiformis* jelly sensory quality.

As shown in Fig. 2, the sensory score of *G. lemaneiformis* jelly is related to the heating time, which initially increased and then decreased. The sensory scores of samples heated for 10 min and 15 min ranged between 65 and 75 points, indicating a satisfactory gel state. The highest score (85 points) was achieved at 20 min of heating, while prolonged heating (30 min) reduced the score due to excessive hardness. These results align with studies demonstrating that extended heating disrupts polysaccharide networks in algal gels, leading to texture degradation (Zhang et al., 2024). An optimal heating time of 15–25 min is therefore recommended for balancing gel integrity and sensory quality.

#### 3.1.3 Effects of vinegar addition on *G. lemaneiformis* jelly sensory quality

**Fig. 3:**
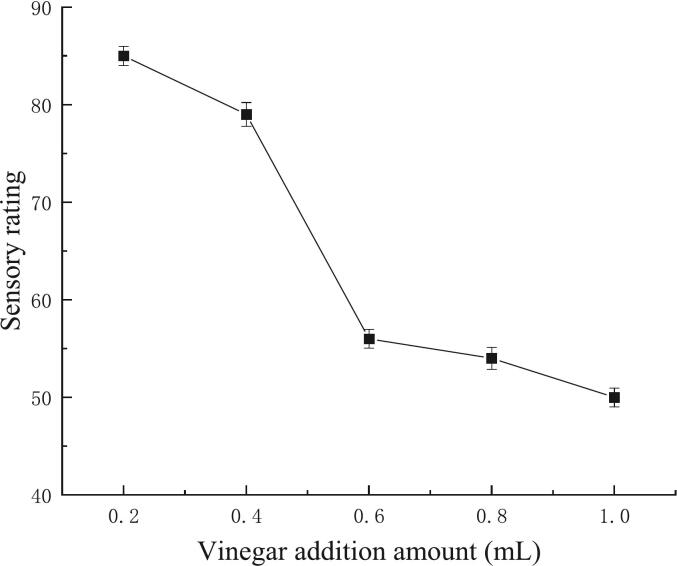
Effects of vinegar addition on *Gracilaria lemaneiformis* jelly sensory quality.

The sensory score decline at high vinegar additions (≥ 0.6 mL) (Fig. 3) reflects pH-driven disruption of the polysaccharide gel network. GLPs form gels through helical aggregation stabilized by hydrogen bonds, which are pH-sensitive (Chen et al., 2024). Excessive acetic acid lowers the pH below 4.0, protonating sulfate groups on GLPs and increasing electrostatic repulsion between helices. This inhibits junction zone formation, leading to a fragmented microstructure and reduced elasticity—consistent with rheological observations in carrageenan gels under acidic conditions (Zhang, Zhang, & Zhang, 2011).

### 3.2 Orthogonal-fuzzy optimization of *g. lemaneiformis* jelly

Through a single factor test, the trend of the effect of solid–liquid ratio, heating time and vinegar addition on product quality was obtained. The superior level of each factor was selected, and the three-factor, three-level orthogonal test was conducted to determine the optimal formula of *G. lemaneiformis* jelly.

#### 3.2.1 Hierarchical weighting analysis of sensory attributes

Based on the initial importance of the sensory evaluation, the ‘0–4 evaluation method’ was used to determine the weight of five people, and the scoring results of the five people are shown in Table 3.

**Table 3:** Evaluation of *Gracilaria Lemaneiformis* jelly weight.

Organoleptic qualities	Sensory scores						
	a	b	c	d	e	score	weight
Color	4	5	6	4	4	23	0.19
mouthfeel	9	8	8	9	10	44	0.36
Organizational status	6	5	6	5	7	29	0.24
flavor	5	6	4	6	3	24	0.2
total	24	24	24	24	24	120	1.0

As shown in Table 3, amongst the 120 points, 23 points are for color, 44 points for taste, 29 points for tissue state and 24 points for flavor, from which the weight coefficients of each factor are obtained. The weights of color, taste, tissue state and flavor were 0.19, 0.36, 0.24 and 0.2, respectively, and the weight of taste accounted for 36 %, which was the most critical index. Furthermore, the weights of color, tissue state and flavor were the same.

#### 3.2.2 Fuzzy sensory evaluation system and matrix development

Five experienced food professionals were selected to evaluate the nine groups of experimental products one by one, and the evaluation results are shown in the table. Each number in Table 4 represents the number of people who have given this rating grade.

**Table 4:** Fuzzy sensory assessment of *Gracilaria Lemaneiformis* jelly weight.

Test number	Color			taste			texture			flavor		
	A1	A2	A3	A1	A2	A3	A1	A2	A3	A1	A2	A3
1	3	2	0	0	1	4	0	0	5	0	2	3
2	1	3	1	0	2	3	0	0	5	0	3	2
3	2	2	1	1	2	2	2	2	1	1	4	0
4	4	1	0	1	3	1	3	1	1	1	3	1
5	4	1	0	2	2	1	4	1	0	1	4	0
6	5	0	0	2	3	0	4	1	0	2	2	1
7	5	0	0	4	1	0	5	0	0	0	5	0
8	0	4	1	1	2	2	0	1	4	1	3	1
9	0	2	3	0	2	3	0	0	5	1	2	2

The data in Table 4 were converted into fuzzy math matrices, and the results are as follows:

$$R_{1}=\left(\begin{array}{ccc} 3/5 & 2/5 & 0/5\\ 0/5 & 1/5 & 4/5\\ 0/5 & 0/5 & 5/5\\ 0/5 & 2/5 & 3/5 \end{array}\right)=\left(\begin{array}{ccc} 0.6 & 0.4 & 0\\ 0 & 0.2 & 0.8\\ 0 & 0 & 1\\ 0 & 0.4 & 0.6 \end{array}\right),$$

$$R_{2}=\left(\begin{array}{ccc} 0.2 & 0.6 & 0.2\\ 0 & 0.4 & 0.6\\ 0 & 0 & 1\\ 0 & 0.6 & 0.4 \end{array}\right)\;R_{3}=\left(\begin{array}{ccc} 0.4 & 0.4 & 0.2\\ 0.2 & 0.4 & 0.4\\ 0.4 & 0.4 & 0.2\\ 0.2 & 0.8 & 0 \end{array}\right),$$

$$R_{4}=\left(\begin{array}{ccc} 0.8 & 0.2 & 0\\ 0.2 & 0.6 & 0.2\\ 0.6 & 0.2 & 0.2\\ 0.2 & 0.6 & 0.2 \end{array}\right)\;R_{5}=\left(\begin{array}{ccc} 0.8 & 0.2 & 0\\ 0.4 & 0.4 & 0.2\\ 0.8 & 0.2 & 0\\ 0.2 & 0.8 & 0 \end{array}\right),$$

$$R_{6}=\left(\begin{array}{ccc} 1 & 0 & 0\\ 0.4 & 0.6 & 0\\ 0.8 & 0.2 & 0\\ 0.4 & 0.4 & 0.2 \end{array}\right)\;R_{7}=\left(\begin{array}{ccc} 1 & 0 & 0\\ 0.8 & 0.2 & 0\\ 1 & 0 & 0\\ 0 & 1 & 0 \end{array}\right),$$

$$R_{8}=\left(\begin{array}{ccc} 0 & 0.8 & 0.2\\ 0.2 & 0.4 & 0.4\\ 0 & 0.2 & 0.8\\ 0.2 & 0.6 & 0.2 \end{array}\right)\;R_{9}=\left(\begin{array}{ccc} 0 & 0.4 & 0.6\\ 0 & 0.4 & 0.6\\ 0 & 0 & 1\\ 0.2 & 0.4 & 0.4 \end{array}\right)$$

#### 3.2.3 Fuzzy logic-based sensory evaluation modeling and optimization

Taking R1 as an example, the result was calculated in accordance with the fuzzy mathematical matrix transformation formula *X* = *W* × *R*, where W = (0.19, 0.36, 0.24, 0.2) is a constant vector.

*X*_1_ = *W* × *R*_1_ = (0.19, 0.36, 0.24, 0.2) × $\left(\begin{array}{ccc} 0.6 & 0.4 & 0\\ 0 & 0.2 & 0.8\\ 0 & 0 & 1\\ 0 & 0.4 & 0.6 \end{array}\right)$ =(0.114, 0.228, 0.648).

The overall assessment findings of the sensory quality of *G. lemaneiformis* jelly from studies in groups 2 to 9 were obtained as follows: *X*_1_ = (0.114, 0.228, 0.648); *X*_2_ = (0.038, 0.378, 0.574); *X*_3_ = (0.284, 0.476, 0.230); *X*_4_ = (0.408, 0.422, 0.160); *X*_5_ = (0.528, 0.390, 0.072); *X*_6_ = (0.606, 0.344, 0.040); *X*_7_ = (0.718, 0.272, 0.000); *X*_8_ = (0.112, 0.464, 0.414); *X*_9_ = (0.040, 0.300, 0.650).

The range analysis demonstrated that the material–liquid ratio (Factor A, *R* = 11.39) exerted the most significant influence on sensory scores, followed by vinegar addition (Factor C, *R* = 10.63), while heating time (Factor B, *R* = 2.15) had the least impact. Despite its secondary role, the optimal level for heating time (B₁: 10 min) was selected based on its markedly higher k₁ value (81.40) compared to B₂ (77.29) and B₃ (79.25). This aligns with the orthogonal optimization principle of *“prioritizing significant factors while selecting optimal levels for minor factors”* to maximize overall quality. Furthermore, the choice of B₁ (10 min) minimized polysaccharide degradation and preserved gel elasticity, consistent with single-factor results where prolonged heating (> 20 min) caused hardening and reduced sensory scores (Section 3.1.2). Consequently, the final optimal combination A₂B₁C₃ (6 % material–liquid ratio, 10 min heating, 0.2 mL vinegar) ensures balanced optimization of both dominant and secondary factors, adhering rigorously to experimental design principles while delivering superior product quality (Table 5).

**Table 5:** Orthogonal experimental results.

Test No.	A (Material–Liquid Ratio)	B (Heating Time)	C (Vinegar Addition)	Sensory Score
1	1	1	1	71.30
2	1	2	2	71.16
3	1	3	3	80.01
4	2	1	2	82.92
5	2	2	3	86.04
6	2	3	1	87.69
7	3	1	3	89.97
8	3	2	1	74.67
9	3	3	2	70.05
*K*_1_	222.48	244.20	233.67	
*K*_2_	256.65	231.87	224.13	
*K*_3_	234.69	237.75	256.02	
*k*_1_	74.16	81.40	77.89	
*k*_2_	85.55	77.29	74.71	
*k*_3_	78.23	79.25	85.34	
R	11.39	2.15	10.63	
Factor Priority	A > C > B			
Optimal Combination	A₂B₁C₃			

#### 3.2.4 Microbiological safety assessment of the final product

Microbiological analysis of the *G. lemaneiformis* jelly was conducted according to national food safety protocols. The total aerobic plate count was 15 CFU/g (≤ 100 CFU/g), coliforms were undetectable (< 1 CFU/g, ≤ 10 CFU/g), molds were absent (< 1 CFU/g, ≤ 20 CFU/g), and yeast count was 10 CFU/g (≤ 20 CFU/g). All microbial parameters complied with the *National Food Safety Standards* of China (GB 4789.2–2016; GB 4789.3–2016), confirming the product's hygienic suitability for consumption (National Health Commission of the People's Republic of China, 2016; National Health Commission of the People's Republic of China & State Administration for Market Regulation, 2022).

## 4 Conclusion

This study successfully optimized the formulation of *G. lemaneiformis* jelly through orthogonal testing integrated with fuzzy mathematics evaluation. The validated optimal parameters (6 % material–liquid ratio, 0.2 mL vinegar, 10 min heating) yielded a product with superior sensory attributes, including smooth texture, compact structure, and preserved algal flavor, while maintaining compliance with Chinese microbiological safety standards (GB 4789.2–2016; GB 4789.3–2016). The methodology not only enhances the industrial potential of seaweed-based gel foods but also aligns with growing consumer demand for minimally processed, nutrient-rich alternatives, demonstrating the efficacy of fuzzy mathematical models in complex food system optimization.

## CRediT authorship contribution statement

**Aoxue Hu:** Writing – original draft, Visualization, Validation, Investigation, Formal analysis, Data curation. **Shengjun Wu:** Writing – review & editing, Supervision, Resources, Project administration, Methodology, Investigation, Funding acquisition, Conceptualization.

## Declaration of competing interest

The authors declare that they have no known competing financial interests or personal relationships that could have appeared to influence the work reported in this paper.

## Data Availability

The authors do not have permission to share data.

## References

[bb0005] Cai B.N., Luo L.X., Zhao X.T., Chen H., Wan P., Huang J.T., Pan J.Y. (2024). Administration of *Gracilariopsis lemaneiformis* polysaccharide attenuates cisplatin-induced inflammation and intestinal mucosal damage in colon-26 carcinoma tumor-bearing mice. Journal of the Science of Food and Agriculture.

[bb0010] Cao M.X., Zhang J.Y., Li P.P., Wang J.G., Mi P., Sui Z.H. (2024). Review on recent advances of *Gracilariopsis lemaneiformis* (Rhodophyta). Algal Research.

[bb0015] Chen H.Y., Zhou Y.C., Liu Y., Huang J.Y., Liu H., Liu C.F., Liu Q.M. (2024). Fermented *Gracilaria lemaneiformis* polysaccharides alleviate food allergy by regulating Treg cells and gut microbiota. International Journal of Biological Macromolecules.

[bb0020] Dang D.Y., Sun S.J. (2023). Research progress on the ecological effects of *Gracilaria lemaneiformis* and the physiological effects of different environments on it. Chemical Design Letters.

[bb0025] Dubois M., Gilles K.A., Hamilton J.K., Rebers P.A., Smith F. (1956). Colorimetric method for determination of sugars and related substances. Analytical Chemistry.

[bb0030] Guo S.J., Yang Y.L., Lin D.X. (2009). Rheological study of polysaccharides in *Gracilaria lemaneiformis*. Food Machinery.

[bb0035] Jung J.S., Joo S.Y. (2021). Antioxidant activities of noni juice extracts using various ethanol ratios and quality characteristics of konjac jelly added with noni juices. Korean Journal of Food Science and Technology.

[bb0040] Kim D.H., Kim S.J., Kim M.R. (2019). Physicochemical properties and antioxidant activities of allulose konjac jelly added with *Enteromorpha prolifera*. Journal of the Korean Society of Food Science and Nutrition.

[bb0045] National Health Commission of the People's Republic of China (2016).

[bb0050] National Health Commission of the People's Republic of China, & State Administration for Market Regulation (2022).

[bb0055] Sen T.L., Min P.C., Kim H., Jung K.H., Woo P.J., Cheol P.J., Young R.D. (2023). *Gracilaria chorda* subcritical water ameliorates hepatic lipid accumulation and regulates glucose homeostasis in a hepatic steatosis cell model and obese C57BL/6J mice. Journal of Ethnopharmacology.

[bb0060] Xie J.Y. (2016). A novel fuzzy mathematical method to the sensory evaluation of wine. Research Journal of Applied Sciences, Engineering and Technology.

[bb0065] Yang X., Li A.Q., Li D., Guo Y.R., Sun L.J. (2021). Applications of mixed polysaccharide-protein systems in fabricating multi-structures of binary food gels—A review. Trends in Food Science & Technology.

[bb0070] Zhang C.L., Zhang J.G., Zhang Y.M. (2011). Research on the production process of natural algae jelly. Journal of Shandong Vocational and Technical College of Commerce.

[bb0075] Zhang Y., Li L., Sun S., Cheng L., Gu Z., Hong Y. (2024). Structural characteristics, digestion properties, fermentation properties, and biological activities of butyrylated starch: A review. Carbohydrate Polymers.

[bb0080] Zolfaghari Z.S., Mohebbi M., Najariyan M. (2014). Application of fuzzy linear regression method for sensory evaluation of fried donut. Applied Soft Computing Journal.

